# Outcome of surgical treatment for proximal long segment post intubation tracheal stenosis

**DOI:** 10.1186/1749-8090-8-35

**Published:** 2013-03-01

**Authors:** Reza Bagheri, Mohammadreza Majidi, Ehsan Khadivi, Alireza Sharifian attar, Azadeh Tabari

**Affiliations:** 1 Cardio - Thoracic Surgery & Transplant Research Center, Emam Reza hospital, Faculty of medicine, Mashhad University of Medical Sciences, Mashhad, Iran; 2 Ghaem Hospital, Faculty of medicine, Mashhad University of Medical Sciences, Mashhad, Iran; 3 Emam Reza hospital, Faculty of medicine, Mashhad University of Medical Sciences, Mashhad, Iran; 4 Endoscopic & Minimally Invasive Surgery Research Center, Ghaem hospital, Faculty of medicine, Mashhad University of Medical Sciences, Mashhad, Iran; 5 Nuclear Medicine Research Center, Faculty of Medicine, Imam Reza Hospital, Mashhad University of Medical Sciences, Mashhad, Iran

**Keywords:** Long segment tracheal stenosis, Surgery, Releasing technique

## Abstract

**Background:**

Post intubation long segment tracheal stenosis is a serious problem which usually requires multiple methods of treatment. The aim of this study was to evaluate the results of surgical treatment in long segment post intubation tracheal stenosis.

**Methods:**

Between 2004 to 2008, 20 patients with proximal long segment tracheal stenosis and resection of over 40% of tracheal length, were analyzed in terms of age, sex, clinical symptoms, etiology of stenosis, length of stenosis and resection, role of suprahyoeid release with bilateral hyoeid bone cutting maneuver, post operative complications and life quality 3 year after surgery.

**Results:**

M/F was 2/5, with the average age of 23.5 ± 0.5 years. Average length of stenosis was 4.2 ± 0.4 cm and the average length of resected segment was 5.2 ± 0.4 cm. Early postoperative complications occurred in 4 patients (20%), 5 patients (25%) had late stenosis and 4 of them were treated with multiple dilation and one patient needed tracheostomy and prolonged T. tube. We didn’t have any mortality. 80% of patients had excellent surgical results in follow up period.

**Conclusion:**

Surgery is the best method of treatment in long and multi segment tracheal stenosis.

## Background

Benign tracheal stenosis is an acquired inflammatory lesion which is mostly due to prolonged intubation and tracheostomy. Considering high prevalence of road accidents and head injuries, necessity of prolonged intubation associated with prolonged ICU stay has been increased, and as a result, these patients usually undergo tracheostomy. In case of lack of supervision in these patients, long and segmental tracheal stenosis occurs that complicates the treatment [1]. Since post intubation tracheal stenosis is transmural, the best treatment is resection and primary tracheal reconstruction. Tracheal releasing maneuvers including release of anterior, posterior portion of trachea and chin to chest stitch are routinely performed for reconstruction. Suprahyoeid release can be used in upper stenoses. For resection of distal lesions, right pulmonary hill releasing technique or right main bronchi cutting and its anastomosis to the left bronchi can be performed [2]. Bilateral hyoid bone cutting maneuver is one of the surgical techniques that used particularly in releasing of the trachea in superior resections which is mostly performed in long and segmental tracheal resection [3]. The aim of this study was to evaluate the result of surgical treatment in 20 patients with long segment post intubation tracheal stenosis and the role of bilateral hyoid bone cutting with suprahyoid release technique to facilitate technical considerations.

## Methods

Between 2004 and 2008, 20 patients with proximal long segment tracheal stenosis underwent this surgical technique which was performed at Qaem Hospital of Mashhad University of Medical Sciences-Iran. Trial registration number for randomised clinical trials: “891016” accepted by local ethics committee of Mashhad University of Medical Sciences. This study was approved by the Ethical committee of Mashhad University of Medical Sciences with this Number: 891016, this study is in compliance with the Helsinki declaration. Inclusive criterias: 1) Resection of long segment of trachea 2) bilateral hyoid cutting and routine releasing maneuver (Suprahyoid releasing maneuver with anterior and posterior release and chin to chest sutures) 3) Three years follow up after surgery.

Exclusive criterias: 1) Tracheal stenosis caused by other etiologies 2) Distal tracheal stenosis 3) Patients who could not be a proper candidate for tracheal resection (inoperable patients who were candidate for other treatment such as stent or T-Tube).

Patients were evaluated in terms of age, sex, clinical symptoms, etiology of stenosis, pre-operative functional parameters, length of resected segment, length of stenosis, additional release of the trachea with this technique, surgical complications and quality of life. Patients were followed up to 3 years after surgery (up to 2011).

Patients were divided into 4 different groups according to the quality of life:

1) Excellent: the patient is able to do all daily activities and do heavy sport activities and they can speak normally.

2) Good: the patient is able to do all daily activities but cannot do heavy sport activities and can speak normally. 3) Moderate: the patient is symptom-free only while resting but not able to do normal activities and can speak normally. 4) Poor: the patient is not able to breathe without T. Tube or tracheostomy and cannot speak normally. These criterias were evaluated every 6 months after the operation and the last evaluation was quality of life. (At least 3 years after surgery). All data were processed by SPSS software, version 11.5.

**Figure 1:**
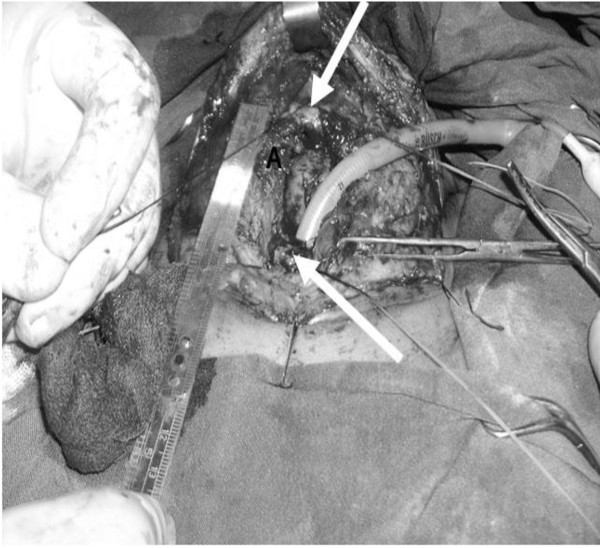
Distance between two end of trachea after posterior and anterior release and suprahyoeid muscle cutting (A).

**Figure 2:**
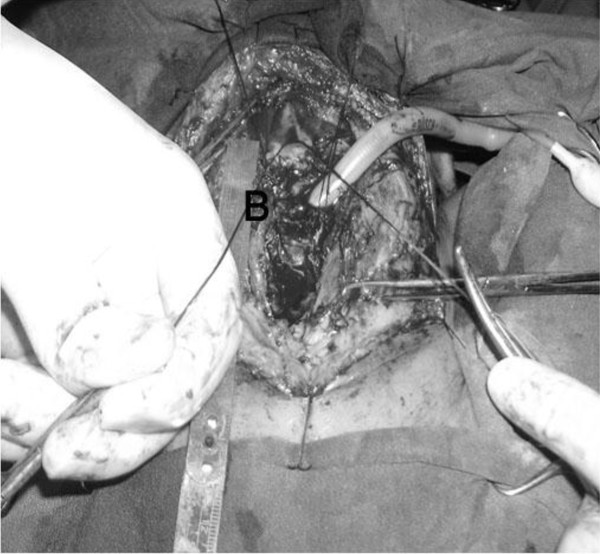
Distance between two ends of trachea after bilateral hyoeid bone cutting (B).

In supine position with hyperextension of neck and collar incision surgery performed with standard technique of tracheal resection. After resection of trachea, posterior and anterior releasing technique and suprahyoid maneuver were performed in all patients prior to anastomosis. Then the interval between trachea was measured after head flexion without tracheal traction (A) (Figure 1). After cutting the two ends of hyoid bone the interval between proximal and distal of trachea was measured (B) (Figure 2). A-B = C was evaluated which is the additional releasing part to the trachea after bilateral hyoid bone cutting. Then, tracheal anastomosis was done with 03 vicryle suture and finally chin to chest stitch was performed. We didn’t use further releasing maneuver such as hilar release. All patients were transferred to ICU.

## Results

This study was accomplished in 20 patients. (Table 1 shows patients’ characteristics). Prolonged intubation due to head injuries was the etiology of tracheal stenosis in all patients. All patients were discharged from ICU with tracheostomy tube (Due to cerebral complications and impairment of consciousness, all the patients underwent tracheostomy) and returned to continue the treatment afterwards. In term of preoperatine function of the patients while all of them had tracheostomy and were unable to breathe without it (group 4). Patients underwent neck CT scan with reconstruction in order to evaluate the length of the trachea and stenosis. Moreover, they underwent rigid bronchoscopy prior to treatment. The mean length of the released segment after bilateral hyoid bone cutting was 1.1 ± 0.4 cm. Resection and anastomosis techniques were done in 16 patients, and due to anterior hyoid bone involvement, posterior tracheal membranous flap technique was performed in other 4 patients.18 patients were extubated and transferred to the ICU, 2 patients were transferred extubated to ICU and tracheostomy was done 3 days afterwards, but was converted to T. Tube due to tracheomalacia 10 days after surgery. Early post operation complications were seen only in 4 patients: two patients with wound infection (10%) and two other patients due to tracheomalacia and collapse of anastomosis area (10%). Patients needed tracheostomy distal to anastomosis suture line and were transferred to ICU. In these patients, removal of the tracheostomy couldn’t be done, so we had to convert tracheostomy to T. Tube. We hadn’t laryngeal dysfunction and hoarseness secondary to recurrent laryngeal nerve injury. Late post operation complication was stenosis that occurred in 5 patients (25%): four of them underwent tracheal dilatation (20%) and one patient underwent multiple tracheal dilation plus T. Tube insertion as a result of post dilatation stenosis recurrence (5%). The characteristics of patients show Table 1.

**Table 1:** Patients characterstics

**Parameter**	
M/F Male/Female	14/6
Mean age	23.5 ± 0.5 M
Mean time between the frist visit and surgery	4.9 ± 0.6 M
Mean Length of Stricture	5.2 ± 0.4 cm
Average Length of stenosis	4.2 ± 0.4 cm

We didn’t have any mortality. Post operative follow up in 3 patients with T. Tube was done. T. Tube was removed in 2 patients 14 and 18 months after surgery, but the third patient had T. Tube in place (5%). Considering patient’s quality of life three years after treatment, following results were noted as: [1] Excellent group including 2 patients (10%) [2] Good group including 14 patients (70%) [3] Moderate group including 3 patients (15%) [4] Poor group including 1 patient (we could not extract the T. Tube after treatment in this group). 17 patients had normal swallowing function after the surgery (85%) and 3 (15%) patients had liquid dysphasia from whom 2 patients recovered 9 months after the surgery and the other patient showed no signs ofrecovery. Table 2 show Considering statistical analysis, factors affecting early and late post operative complications.

**Table 2:** show considering statistical analysis, factors affecting early and late post operative complications

**Risk factors**	**Late post operation complication (tracheal stenosis) N(%)**		**p.value**
Sex			NS
Male	3(15)	11(55)	
Female	2(10)	4(20)	
Age (mean)	22.20	22.40	
Infection of wound			
Yes	2(10)	0(0)	
no	3(15)	15(75)	
Tracheomalacia	Yes	no	
Yes	1(5)	1(5)	
No	4(20)	14(70)	
Length of stenosis (mean)	3.99 ≥ cm	4 ≤ cm	0.023

## Discussion

The blood supply of extrathoracic trachea is from 3 nutritive branches of inferior thyroid artery therefore, tracheal ischemia leads to inflammation, granulation and eventually tracheal transmural stenosis [2]. One of the most common reasons of this complication is using high-pressure cuffed endotracheal tube or tracheostomy tube, which leads to increment of pressure in the area, as well as ischemia and tracheal stenosis in prolonged usage. The high incidence of tracheal stenosis in the site near the tip of the intubations tube and the site of tracheostomy, other than the endotracheal tube cuff area, results in long and multisegmental tracheal stenosis in patients who underwent prolonged mechanical ventilation [2]. Stridor is one of the prevalent signs in patients with tracheal stenosis, especially in those with the history of intubations in their past medical history. Some studies mentioned that even 24 hours of intubation is enough for this complication to occur. It should be noted that in some cases patients may be intubated long time ago and they would not remember the occasion. On the other hand, some studies showed that patients can become symptomatic even one year after inubation [4,5]. Patients with stridor and the history of prolonged intubation should undergo general anesthesia before making any therapeutic decisions. Consequently, no additional tracheal injuries will occur due to urgent tracheostoma in an inappropriate setting [6]. In this study, all patients underwent temporary tracheostomy in other words, and after referring to our ward, they underwent general anesthesia and were evaluated by rigid bronchoscopy. Therefore, the expansion of the stenosis and the anatomy of trachea were precisely assessed. The treatment of post-intubation tracheal stenosis has been a therapeutic challenge for years. Various techniques have been discussed as different treatments including repeated dilatation, laser therapy, cryosurgery and surgery. The first three aforementioned techniques have some problems like high failure and the need for repeating the procedure; therefore, surgery is the treatment of choice in post-intubations tracheal stenosis [7]. The resection of long and multi segmental tracheal stenosis is one of the therapeutic difficulties which mostly affects the primary reconstruction of these patients. Grillo et al. (1995) studied 503 patients with tracheal stenosis who underwent 521 surgeries (tracheal or laryngotracheal surgeries). The result of treatment in 93.7% of patients was divided into 2 groups of good and excellent, so they mentioned that the preferable treatment is surgical resection and reconstruction of affected segment [2]. In 2007, Babarro and colleagues performed a study on the long tracheal resections. They pointed out that surgical treatment is the method of choice in these cases. They also claimed that the T. Tube was inserted in patients with long resection at the end of the surgery. Besides, they mentioned that the suprahyoid muscle releasing maneuver and bilateral hyoid bone cutting are useful to prevent the traction in resection of proximal stenosis [8]. A study by Marulli et al. (2006) reported that one time resection and primary reconstruction can be done for long stenosis with laryngeal involvement in benign stenosis, having a good long time result (approximately 93.3% for the 2 groups of good and excellent) [7]. In addition, a study by Wynn R and colleagues (2004) revealed that the results of surgery and primary reconstruction in the treatment of tracheal stenosis have high success rate [9]. In a study on the resection techniques of proximal long segment tracheal stenosis (more than 40% of tracheal length), Soon et al. emphasized on the releasing maneuvers especially suprahyoid muscle releasing maneuver and bilateral hyoid bone cutting. They also mentioned that hyoid bone cutting in limited resections was not necessary [3]. Tracheal surgery has early and late complications. Some of the early complications are as follows: dehiscence of anastomosis resulting from excessive traction in suturing site, wound infection, respiratory tract edema. The most prevalent late complication is tracheal stenosis recurrence due to granulation tissue formation. As a matter of fact, dehiscence of anastomosis is the most dreaded, and late granulation tissue formation and wound infection are the most prevalent complications [2]. The important point was that the dehiscence of anastomosis site resulting from tension in suture line occurred because of performing the tracheal releasing techniques.

Based on the fact that one of the complications in tracheal surgery is post operative granulation tissue formation which is a result of the proportional traction in the anastomosis site, choosing the appropriate suture string is important. Therefore, the sutural knot should be formed extratracheally to prevent the granulation tissue formation. In a study by Behrend et al., 3 types of absorbable suture strings (poly propylene, polydio, polyglactin) were used in tracheal surgery. They revealed that the results were similar in all 3 groups, but also mentioned that the sutural string should be with high tension ability and shouldn’t be absorbed in less than 6 months. They also mentioned that techniqual matters especially tension are more effective than choosing the suture string in post operative results [10]. Nowadays endoscopic treatment of post-intubation benign stenosis has its own adherents. Gulluccio et al. (2009) reported that repeated dilatation, stenting or laser therapy have a role in treatment of simple and short segment post intubation tracheal stenosis, but surgery is the method of choice in long and multi segmental tracheal stenosis [11]. Moreover, in a study by Nouraei SA et al. (2007) endoscopic treatments were assessed as effective procedures in post-tracheostomy limited stenosis. They claimed that surgery is the method of choice in long and multi segment tracheal stenosis [12]. Cavaliere S et al. (2007) also pointed out that endoscopic treatments can be used in some specific patients as a supplementary procedure besides surgery, or as a particular method in post operative stenosis [13]. In this study endoscopic treatments (repeated dilatation) were performed in post operative stenosis recurrence as a supplementary procedure, but not as the first line treatment. Because of long segment stenosis, surgery was the first choice of treatment in these patients. One of the other therapeutic methods in post operative recurrent stenosis is applying polyflex stents.

A study by Bagheri et al. (2004) revealed that the usage of polyflex stent is inappropriate due to the complications that occurred after a few months. Therefore, the stent should be removed, which can lead to granulation tissue formation and dramatic stenosis.

Eventually they recommended T. Tube insertion in an inoperable complex stenosis [14].

## Conclusion

Considering high prevalence of post-intubation tracheal stenosis, especially the long segment types, good therapeutic results can be achieved by performing a multi disciplinary strategy (surgery, endoscopic treatment and combined treatment). Despite few numbers of patients in this study and the need for further clinical studies for definitive conclusion based on our results.

## Competing interests

The authors declare that they have no competing interests.

## Author’s contributions

RB, MM, EK, ASattar, AT, RB carried out date collection and writing, date analysis and study design. EK, performed study design data collections and writing. ASA carried out data collections. AT carried out study design data collections and writing. All authors read and approved final manuscript.

## Authors’ information

Co-corresponding author: Reza Bagheri, Mohammadreza Majidi, Ehsan Khadivi and Azadeh Tabari and corresponding author Alireza Sharifian attar.
